# PPAR Gamma agonists regulate tobacco smoke-induced toll like receptor 4 expression in alveolar macrophages

**DOI:** 10.1186/1465-9921-15-28

**Published:** 2014-03-11

**Authors:** Yan Yin, Gang Hou, Erran Li, Qiuyue Wang, Jian Kang

**Affiliations:** 1 Institute of Respiratory Disease, The First Affiliated Hospital of China Medical University, Shen Yang City, China

**Keywords:** Peroxisome proliferator-activated receptor γ, Cigarette smoke, Toll-like receptors, Alveolar macrophages, Nuclear factor-kappa B

## Abstract

**Background:**

Peroxisome proliferator-activated receptor-gamma (PPARγ) is a ligand-activated transcription factor that exerts multiple biological effects. Growing evidence suggests that PPARγ plays an important role in inflammation; however, the effects of this transcription factor on the inflammation caused by smoking are unclear.

**Methods:**

We measured the expression of inflammatory cytokines (leukotriene B4, LTB4 and interleukin 8, IL-8), PPARγ and toll-like receptors (TLR2 and TLR4) in alveolar macrophages (AMs) harvested from rats exposed to cigarette smoke (CS) for 3 months *in vivo*. Some of the rats were pre-treated with rosiglitazone (PPARγ agonist, 3 mg/kg/day, ip), rosiglitazone (3 mg/kg/day, ip) + BADGE (bisphenol A diglycidyl ether, a PPARγ antagonist, 30 mg/kg/day, ig), or BADGE alone (30 mg/kg/day, ig). We also measured the expression of PPARγ, TLR2, TLR4 and nuclear factor-kappaB (NF-κB) in AMs gained from normal rats, which exposed to 5% CSE (cigarette smoke extract) for 12hrs, respectively pretreated with PBS, rosiglitazone (30 uM), rosiglitazone (30 uM) + BADGE (100 uM), 15d-PGJ2 (PPARγ agonist, 5 uM), 15d-PGJ2 (5 uM) + BADGE (100 uM), or BADGE (100 uM) alone for 30 min *in vitro*.

**Results:**

*In vivo,* rosiglitazone counteracted CS-induced LTB4 and IL-8 release and PPARγ downregulation, markedly lowering the expression of TLR4 and TLR2. *In vitro,* both rosiglitazone and 15d-PGJ2 inhibited CS-induced inflammation through the TLR4 signaling pathway.

**Conclusions:**

These results suggest that PPARγ agonists regulate inflammation in alveolar macrophages and may play a role in inflammatory diseases such as COPD.

## Background

Chronic obstructive pulmonary disease (COPD) is a chronic inflammatory disease of the airways that is characterized by progressive limitations in airflow. Cigarette smoking is one of the most important risk factors for COPD and persistent airway inflammation [1]. Eliminating the inflammation caused by cigarette smoke (CS) is a goal of COPD treatments. Peroxisome proliferator-activated receptor gamma (PPARγ), a member of the nuclear hormone receptor superfamily [2], has been identified in lung tissue and the cells associated with inflammation in the lung [3-5]. Therefore, PPARγ agonists may be the next choice for COPD treatment. Recent studies have shown that PPARγ expression is reduced in the skeletal muscles, airways, and alveolar macrophages (AMs) of individuals suffering from chronic pulmonary diseases. Recently, the studies have shown increased PPARγ expression in the bronchial epithelial cells of asthma patients, but decreased PPARγ expression in allergic inflammation and acute lung injury induced by LPS [6-8]. Moreover, thiazolidinediones exert anti-inflammatory effects by activating PPARγ and downregulating nuclear factor-κB (NF-κB) [9,10]. These results have led to increasing interest in PPARγ and its involvement in a variety of disease states, including COPD.

While PPARγ agonists exhibit anti-inflammatory effects, the effect of these molecules in CS-induced chronic inflammation is largely unknown. AM-mediated inflammation plays a critical role in the development of COPD [11,12], and the engagement of Toll-like receptors (TLRs) can trigger AMs to produce inflammatory mediators [13]. Some anti-inflammatory mediators reduce airway inflammation through the TLR2/TLR4 pathway [14], but little is known about the interaction between the TLR2/TLR4 pathway and the anti-inflammatory PPARγ pathway. Given these considerations, we sought new insight into the role of PPARγ agonists in preventing chronic airway inflammation and impairing the AM response to CS. To gain a better understanding of the PPARγ mechanism of action in AMs, we also investigated the effects of PPARγ agonists on the expression of TLR2, TLR4 and NF-κB. Additionally, we investigated whether BADGE, a PPARγ antagonist, attenuates the protective effect of PPARγ agonists.

## Methods

### Animals and experimental design

All of the experiments were conducted in accordance with ethical committee guidelines. As shown as Additional file 1: Figure S1, male Wistar rats (Laboratory Animal Center, China Medical University) with a weight range of 170–220 g were randomly placed into one of five groups of 12 rats: sham, CS, PPARγ agonist rosiglitazone (ROSI), PPARγ antagonist BADGE (BADGE), and ROSI + BADGE (RB). The rats were sacrificed by exsanguination before excision of the lungs at the end the 12th week. The right upper lobes were removed and stored at -80°C.

### Tissue preparation and morphometric analyses

The middle lobes of the right rat lungs, which were not lavaged, were embedded in paraffin blocks, and sectioned at 4-μm thickness for conventional HE staining. The measure of lung tissue morphology was determined by light microscopy at a magnification of × 200. At least two nonconsecutive slides per block were analysed for the following: (i) mean linear intercept (MLI), which was a measure of interalveolar wall distance, defined by the total length of the cross-line divided, by the numbers of alveolar wall intersecting the test lines; (ii) mean alveolar number (MAN), which was an indicator of alveolar density calculated by counting the numbers of alveoli in each field.

### Isolation and culture of alveolar macrophages

The left lungs were infused with 2 ml PBS for 4 times. The bronchoalveolar lavage fluid (BALF) was centrifuged for 10 min at 1000 r/min and 4°C. The pellets obtained from the BALF were washed twice with cold Phosphate Buffered Saline (PBS) and resuspended in PBS at 1 × 106 cells/ml. The cells were then incubated in 6-well plates in 2 ml RPMI-1640 medium with 10% fetal calf serum (FCS). All of the nonadherent cells were removed by washing with PBS. We used a previously described modified H&E staining method [15] to identify alveolar macrophages (AMs) based on morphology. The purity of the cell suspension was >95%.

### Phagocytosis and viability of alveolar macrophages

AMs were harvested from the BALF of different groups, and 2 × 10^5^ AMs/well were cultured in RPMI-1640 for 3 hrs or 24 hrs. Phagocytosis was measured with the neutral red uptake method described in previous article [16]. All of the nonadherent cells were removed by washing with PBS. The adherent cells were incubated in 100 μL of RPMI-1640 and 100 μL of neutral red (0.072%) reagent for 4 hrs. The plates were then washed to remove the excess dye and blotted dry. The incorporated dye was re-suspended in ethanol (50%) containing glacial acetic acid (1%). Subsequently, the absorbance at OD490 was read using a spectrophotometer. The absorbance (A) was translated into a phagocytosis ratio to make comparisons: phagocytosis ratio = test A/normal control A × 100%.

For the metabolic activity assays *in vivo*, AMs gained from each group were cultured in 96-well plates at a density of 1 × 10^5^ cells/well. AMs were stimulated with 5% CSE in RPMI-1640 with 10% FCS for 4 hrs in a humidified atmosphere of 5% CO_2_ and 37°C. After treatment, the medium was discarded and 200 μL of DMEM containing 20 μL of MTT (methylthiazolyldiphenyl-tetrazolium bromide, 5 mg/mL, pH = 7.4) reagent was added to each well. The cells were incubated for 4 hrs at 37°C. The medium was again discarded, DMSO was added to each well, and the MTT activity was measured at an optical density of 570 nm. The absorbance (A) was translated into a viability ratio to make comparisons: viability ratio = test A/normal control A × 100%.

For the metabolic activity assays *in vit*ro, AMs gained from normal rats were stimulated with 1% CSE (cigarette smoke extract), 5% CSE and 10% CSE individually for 6 hrs. The cells were pretreated with PBS, ROSI (30 μM), ROSI (30 μM) + BADGE (100 μM) (BADGE was administered 30 min before ROSI), 15-deoxy-Δprostaglandin J2 (15d-PGJ2, a natural ligand of PPARγ, 5 μM) or 15d-PGJ2 (5 μM) + BADGE (100 μM), (Sigma-Aldrich Corporation, St. Louis, MO, USA) for 30 min before being treated with different concentration of CSE.

### Immunofluorescence staining of TLR2 and TLR4 in AMs

The sections was incubated with 5% BSA in PBS at room temperature for 60 min, and then incubated with primary rabbit anti-rat TLR4 and anti-rat TLR2 antibodies (1:300, Santa Cruz, CA, USA) at 4°C overnight. The primary antibody was detected with biotinylated anti-rabbit Ig at a 1:200 dilution. Bound antibody was visualized with ABC peroxidase. Images were obtained with a confocal microscope (Olympus, Japan). The images were quantified by analyzing the sum of the staining with Metamorph DP10 (Olympus, Japan). The negative controls that received PBS were run in parallel. The endogenous peroxidase activity of the AMs treated with cigarette smoke extract *in vitro* was detected using the same protocol described above.

### Stimulation of AMs with CSE and the culture of AMs with ROSI, BADGE and 15dPGJ2

The CSE was prepared as follows: 2 filtered cigarettes (3R4F), each described by the manufacturer as containing 0.73 mg of nicotine, 9.4 mg of tar, and 12.0 mg of CO, were bubbled through 20 ml serum free RPMI-1640 medium with a mechanical vacuum pump. The extract was filtered through a 0.45-μm filter (Millipore, Bedford, MA, USA) to remove bacteria and particles. CSE concentration was evaluated by measuring the optical density at 502 nm, and diluted to O.D. = 0.17 ± 0.03. This solution was considered 10% CSE.

The AMs harvested from the normal rats. The normal AMs were stimulated with 1% CSE, 5% CSE and 10% CSE individually for 12 hrs, after which we analyzed the changes in TLR2, TLR4 and PPARγ expression, the release of LTB4 and IL-8 into the cell culture supernatant and the viability of the AMs. The cells pretreated with ROSI (30 μM), ROSI (30 μM) + BADGE (100 μM) (BADGE was administered 30 min before ROSI), 15d-PGJ2 (5 μM), 15d-PGJ2 (5 μM) + BADGE (100 μM), or PBS for 30 min before being treated with 5% CSE for 12 hrs. We further investigated the above-mentioned parameters in the presence or absence the NF-κB inhibitor PDTC (10 μmol/L) (Sigma-Aldrich Corporation, St. Louis, MO, USA). In addition, We detected the secretions of LTB4 and IL-8 into the cell culture supernatant, when the cells co-treated with anti-mouse specific antibody for TLR4 (eBioscience, San Diego, CA, USA) and 5% CSE.

### Real-time PCR analysis for measurement of TLR2, TLR4 and PPARγ

Total RNA was prepared from AMs, using Trizol according to the manufacturer’s instructions. PCR was carried out with the One-Step qRT-PCR kit (TaKaRa Co, Dalian, China) performed on an ABI PRISM 7500 instrument (ABI, Foster City, CA, USA.), following the manufacturer’s instructions. Primers for PPARγ, TLR2, TLR4 and β-actin using gene-specific primers (Table 1). The PCR parameters were initial denaturation at 94°C for 2 min, followed by 40 cycles of 94°C for 30 s and 72°C for 60s. Gene expression was quantified using a comparative critical threshold (CT) method as described previously [17].

**Table 1:** **Primers for gene-specific reverse transcription and real-time polymerase chain reaction (*in vivo *** **test and *in vitro *** **test)**

**Gene**	**Forward primer**	**Reverse primer**
PPARγ	ATTCTGGCCCACCAACTTCGG	TGGAAGCCTGATGCTTTATCCCCA
TLR2	GTCCATGTCCTGGTTGACTGG	GATACCACAGCCCATGGAAAT
TLR4	GAGCCGGAAAGTTATTGTGG	AGCAAGGACTTCTCCACTTTCT
β-actin	GCCAACCGTGAAAAGATG	CCAGGATAGAGCCACCAAT

### Flow cytometric analysis of the surface expression of TLR2 and TLR4 in AMs

Frozen AMs were washed with PBS and pelleted by centrifugation (800 rpm for 5 min at 4°C). The samples were resuspended at 1 × 10^6^ cells/2 ml RPMI-1640 medium, after which a fluorescein isothiocyanate (FITC)-conjugated anti-rat TLR2 mAb and a TLR4 mAb were added to the cells for 60 min on ice, as instructed by the manufacturer. The cells were then analyzed with FACS and Cell Quest software.

### ELISA for measurement of IL-8 and LTB4 in Bal fluid and culture supernatants

The expressions of interleukin-8 (IL-8) and leukotriene B4 (LTB4) in BAL fluid and culture supernatants were determined using the QuantiGlo ET-1 Immunoassay System (BD Biosciences, Bedford, MA), according to the manufacturer’s protocol. BCA (bicinchoninic acid) protein assay was used to correct the homogenate supernatants of 50 mg lung tissues in the different groups as measured by enzyme-linked immuno sorbent (ELISA).

### Western blot analysis for PPARγ, TLR4, NF-κB

20 μg of isolated total protein was subjected to electrophoresis on a 10% polyacrylamide (PAGE) gel and transferred onto a nitrocellulose membrane by electroblotting. The membrane was blocked for 1 hrs at room temperature with blocking solution. The blot was then incubated overnight at 4°C with rabbit anti- PPARγ, anti-TLR4, anti-I-κB or anti-P65 antibody (1:500; Santa Cruz Biotechnology, Santa Cruz, CA, USA). After three washing steps, the membrane was incubated with secondary antibody (1:2000 dilution) for 2 hrs at room temperature. Bound complex was detected using enhanced chemiluminescence (Amersham Biosciences, NJ, USA). Densitometric techniques were performed to quantify the protein band densities (Metamorph/Evolution MP 5.0/B × 51), which were expressed as mean relative densitometric units.

## Results

### ROSI attenuated CS induced histological changes

CS exposure induced airway inflammation accompanied by focal emphysema. First, the airway wall may have been thickened by inflammatory cell infiltrates or structural changes. Second, the airway lumen was occluded by mucous secretions. Third, alveolar attachments became disrupted as a result of emphysema. The sham group displayed no discernible histological changes. The ROSI exhibited a protective effect on emphysematous changes and airway inflammation induced by CS exposure. And these effects were partially attenuated by BADGE. Moreover, there was no difference between CS group and BADGE groups. (Additional file 1: Figure S2, Figure S3 and Table S1).

### ROSI reduced the number of inflammatory cells present in the BALF after CS exposure

As shown in Table 2, CS generally increased the counts of total cells, neutrophil %, lymphocytes % and the number of AMs, and decreased AMs % count. Compared to CS group, ROSI- treatment induced the reductions of the counts of total cells and neutrophiles %. ROSI-treatment tended to attenuate the reduction of AMs % induced by CS exposure, however, none of differences were statistically significant (*P* = 0.06 > 0.05). There was no significant difference between the BADGE- treated and the CS only groups.

**Table 2:** BAL Fluid cytology of rats of each group

**Group**	**Sum (× 106/L)**	**Lymphocytes %**	**Macrophages %**	**Neutrophils %**
Sham	3.8 ± 1.44	3.8 ± 1.63	88.5 ± 9.43	2.91 ± 1.92
CS only-exposed	13.4 ± 3.29**	9.6 ± 3.45**	66.8 ± 10.66**	20.4 ± 9.44**
ROSI-treated	9.3 ± 2.66**##	6.7 ± 2.20*	76.9 ± 6.34**	11.5 ± 3.14**#
BADGE-treated	13.4 ± 3.37**	9.07 ± 3.16**	68.5 ± 10.16*	20.5 ± 8.66**
RB-treated	11.5 ± 2.06**##	7.8 ± 2.69**	69.1 ± 7.06**	19.9 ± 4.16**

Data represent the mean ± SD (*n* = 6). *P < 0.05 and **P < 0.01 compared with the sham group; #*P* < 0.05 and ##*P* < 0.01 compared with the CS only-exposed group.

### Effects of PPARγ ligands on the phagocytosis and viability of AMs

**Figure 1:**
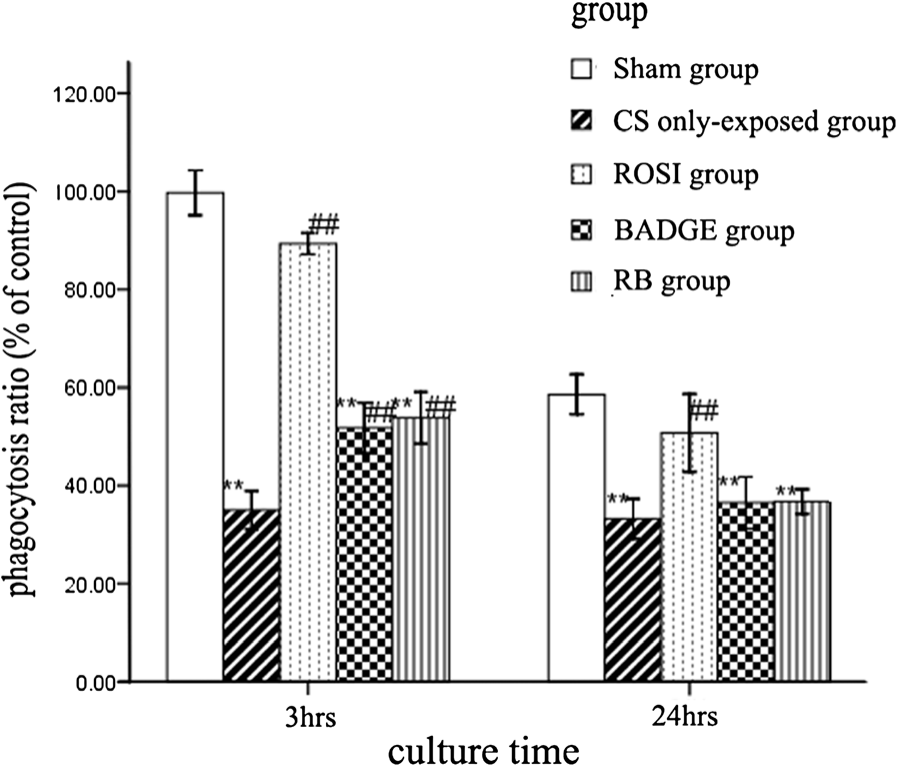
**Phagocytosis by alveolar macrophages from each group.** Macrophages were harvested from the BAL fluid of different groups and were cultured in media for 3 and 24 hrs. The results are presented as mean ± SD (n = 6). *P < 0.05 and **P < 0.01 compared with the sham group; #P < 0.05 and ##P < 0.01 compared with the CS only-exposed group.

**Figure 2:**
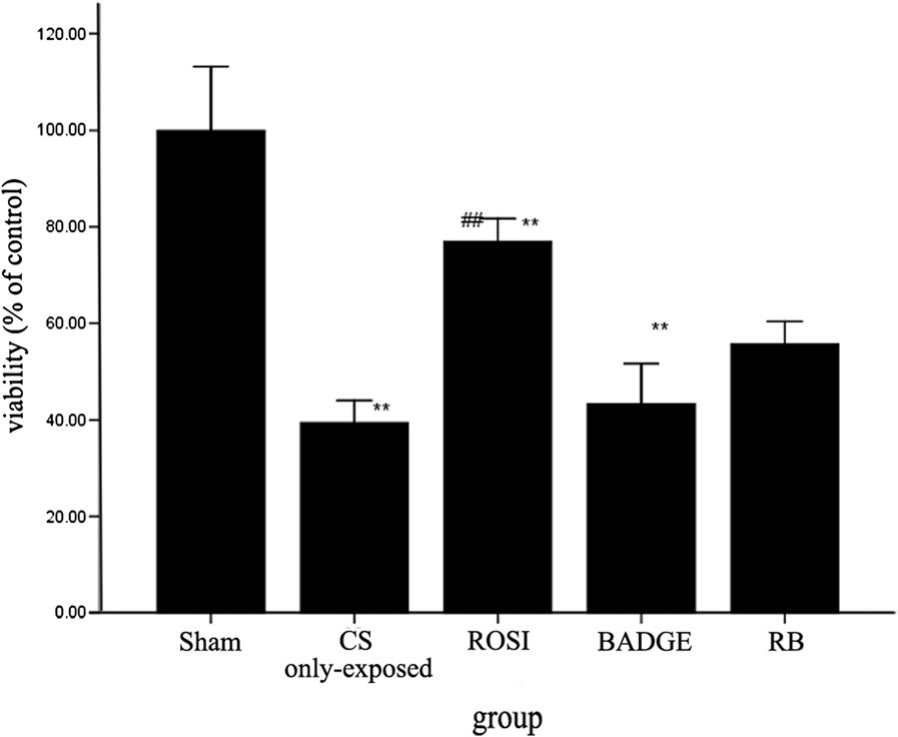
**The AMs metabolic viability of each group.** Macrophages were harvested from BAL fluid of different groups and were treated with 5% CSE for 4 hrs. The results are presented as mean ± SD (n = 6). *P < 0.05 and **P < 0.01 compared with the sham group; #*P* < 0.05 and ##*P* < 0.01 compared with the CS only-exposed group.

*In vivo*, the AMs from the groups exposed to CS exhibited decreased phagocytosis (Figure 1) and viability (Figure 2). The AM-mediated phagocytosis at 3 hrs and 24 hrs showed similar changes: the ROSI-treated group showed an increase in phagocytosis and metabolic viability compared with the CS group, while BADGE partially blocked the ROSI-induced recovery in both phagocytosis and cell viability. The effects of the combined ROSI plus BADGE treatment on phagocytosis and cell viability were similar to the effects of treatment with BADGE alone.

**Figure 3:**
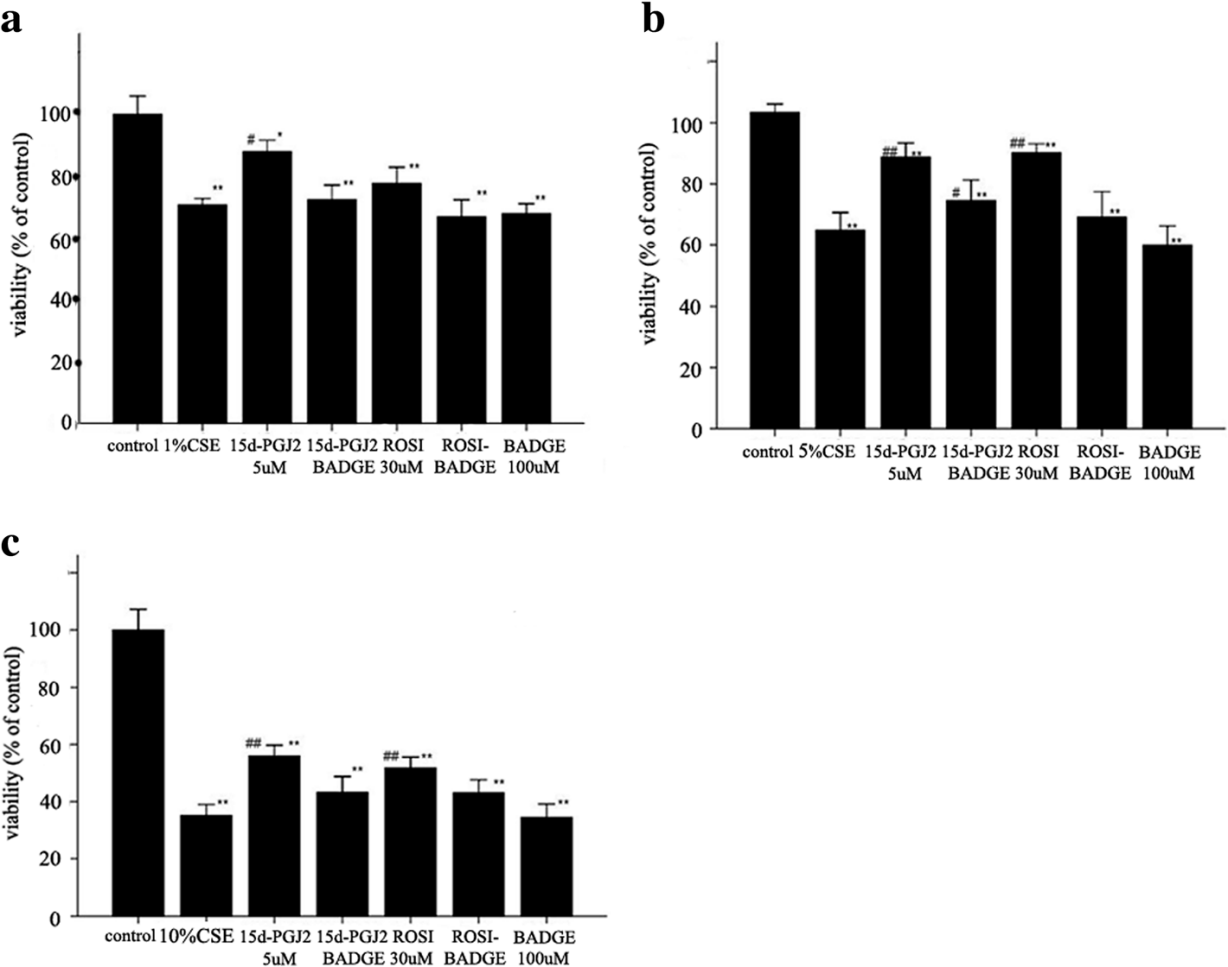
**The metabolic viability test *in vitro*.** AMs gained from normal rats were stimulated with 1% CSE **(a)**, 5% CSE **(b)** and 10% CSE **(c)** individually for 4 hrs. The AMs in response to different drug pretreatment interventions. The results are presented as mean ± SD (n = 3). *P < 0.05 and **P < 0.01 compared with the control group; #*P* < 0.05 and ##*P* < 0.01 compared with the CSE group.

Likewise, the AMs in the primary culture presented similar results *in vitro*. Both 15d-PGJ2 and ROSI attenuated the decrease in cell viability induced by 1%, 5% and 10% CSE. And pre-treatment with BADGE counteracted the protective effect of 15d-PGJ2 and ROSI *in vitro* (Figure 3).

### PPARγ ligands decreased CS-induced inflammatory cytokine production

**Figure 4:**
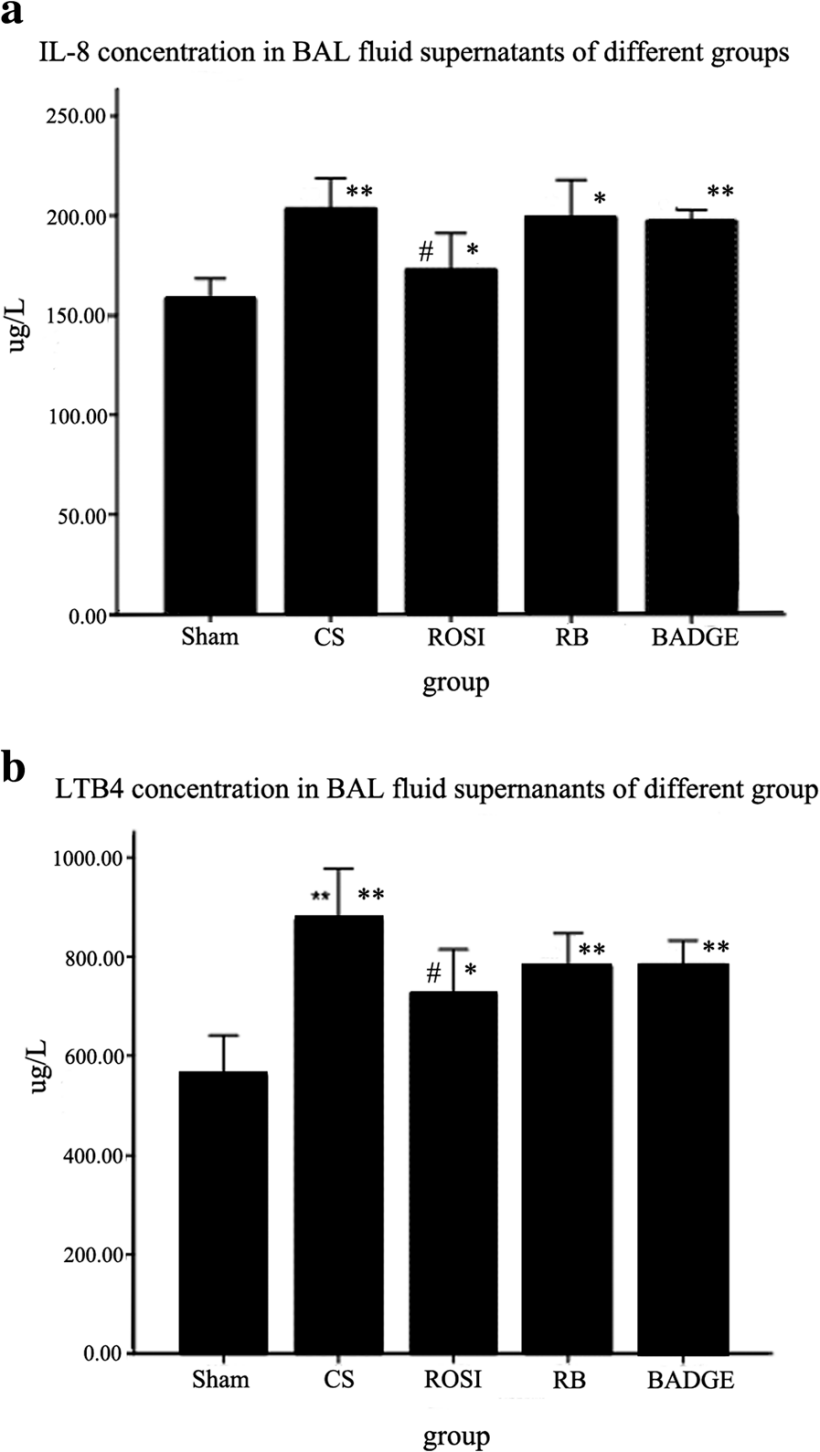
**The concentrations of IL-8 (a) and LTB4 (b) in BAL fluid in different groups were measured by ELISA.** The results are expressed as the mean ± SD (n = 6). *P < 0.05 and **P < 0.01 compared with the sham group; #*P* < 0.05 and ##*P* < 0.01 compared with the CS only-exposed group.

*In vivo*, CS exposure increased focal emphysema as well as IL-8 and LTB4 levels (Figure 4) in the BAL fluid compared to the sham group. Treatment with ROSI decreased the secrections of IL-8 and LTB4 induced by CS, and co-treatment with BADGE weakened above effects of ROSI on cytokines (LTB4 and IL-8).

**Figure 5:**
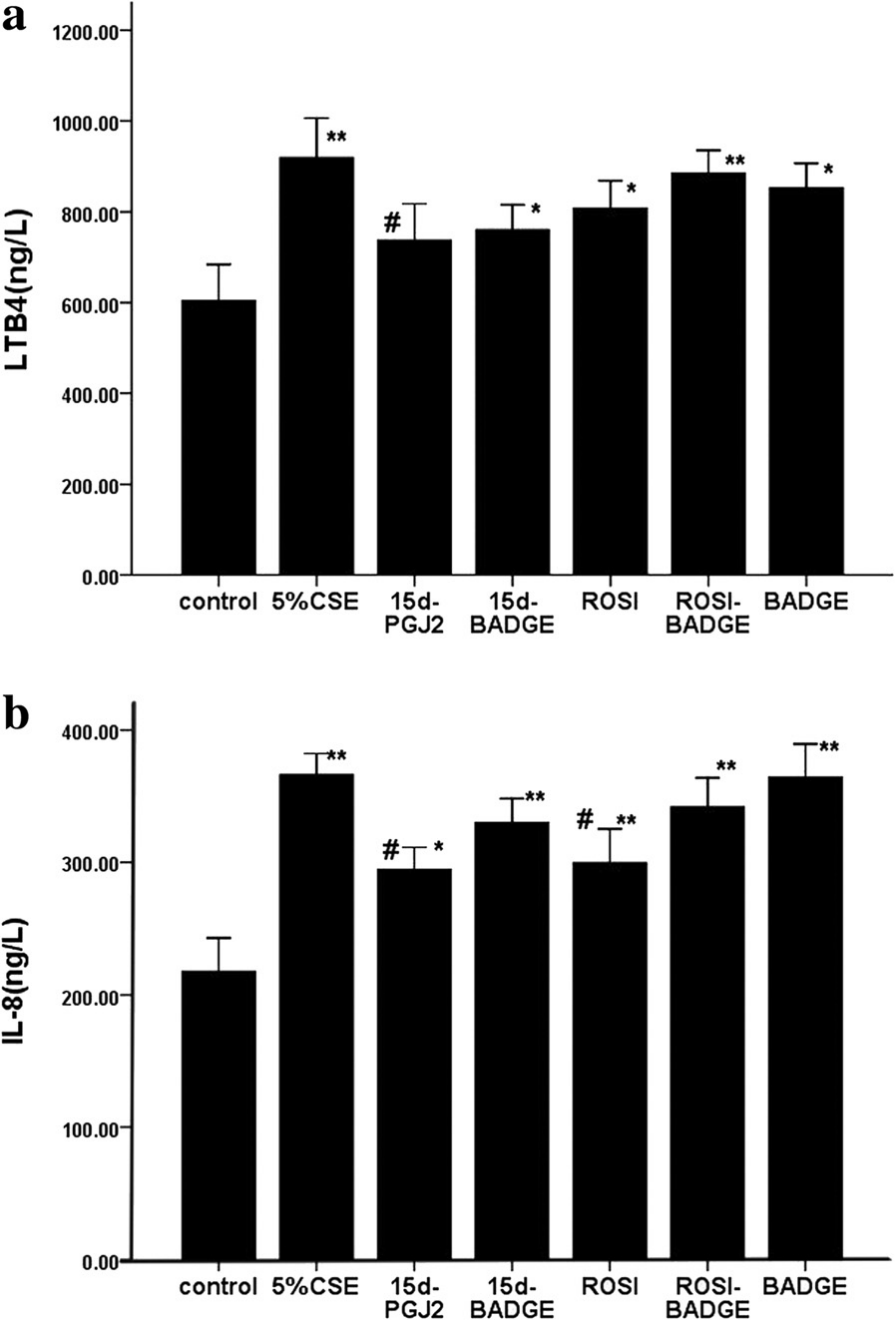
**Cell Supernatants were harvested to measure LTB4 (a) and IL-8 (b) by ELISA.** The results are expressed as the mean ± SD (n = 3). *P < 0.05 and **P < 0.01 compared with the sham group; #*P* < 0.05 and ##*P* < 0.01 compared with the CSE group.

*In vitro* test*,* similar to the results *in vivo,* 5% CSE exposure increased significantly the IL-8 and LTB4 secretions by AMs. ROSI and 15d-PGJ2 attenuated the CSE-induced releases of LTB4 and IL-8 (Figure 5).

### Involvement of TLR4 in CS-induced inflammatory cytokine production

**Figure 6:**
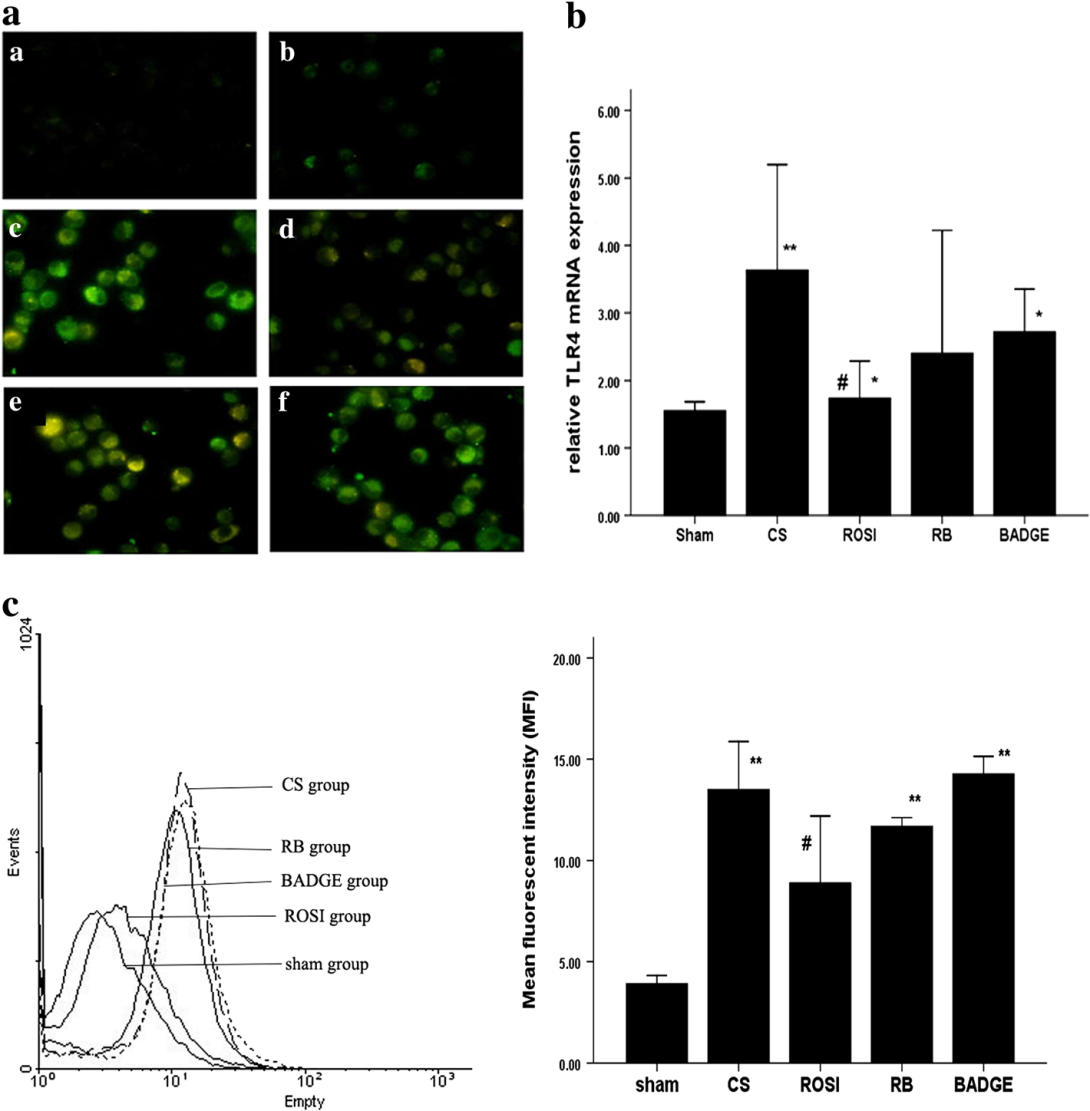
**Expression of TLR4 in AMs gained from each group. a**: Immunofluorescence of AMs from each group of rats with antibodies to TLR4 (green). Representative TLR4 expression was shown in the negative control group (a), the sham (b), CS (c), ROSI (d), RB (e) groups and BADGE (f). Shown are representative images of five rats in each group; **b**: mRNA expression of TLR4 in AMs. The data are representative of six rats in each group. **c**: representative flow cytometry histogram showing surface protein expression of TLR4 in AMs. The data are representative of six rats in each group. The results are presented as mean ± SD. *P < 0.05 and ** *P* < 0.01 compared with the sham group; #*P* < 0.05 and ##*P* < 0.01 compared with the CS group.

**Figure 7:**
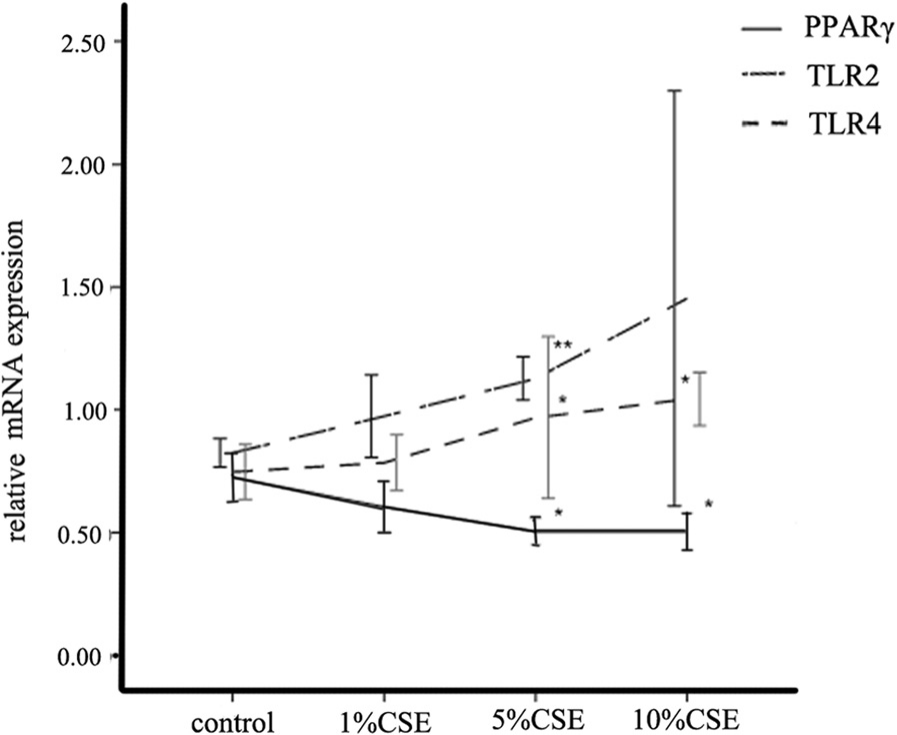
**The expressions of mRNA of TLR2, TLR4 and PPARγ in AMs.** AMs extracted from normal rats and were treated with different concentrations of CSE for 12 hrs *in vitro*. The results are expressed as the mean ± SD (n = 3). The mRNA was determined by real-time PCR. *P < 0.05 and **P < 0.01 compared with the control group.

**Figure 8:**
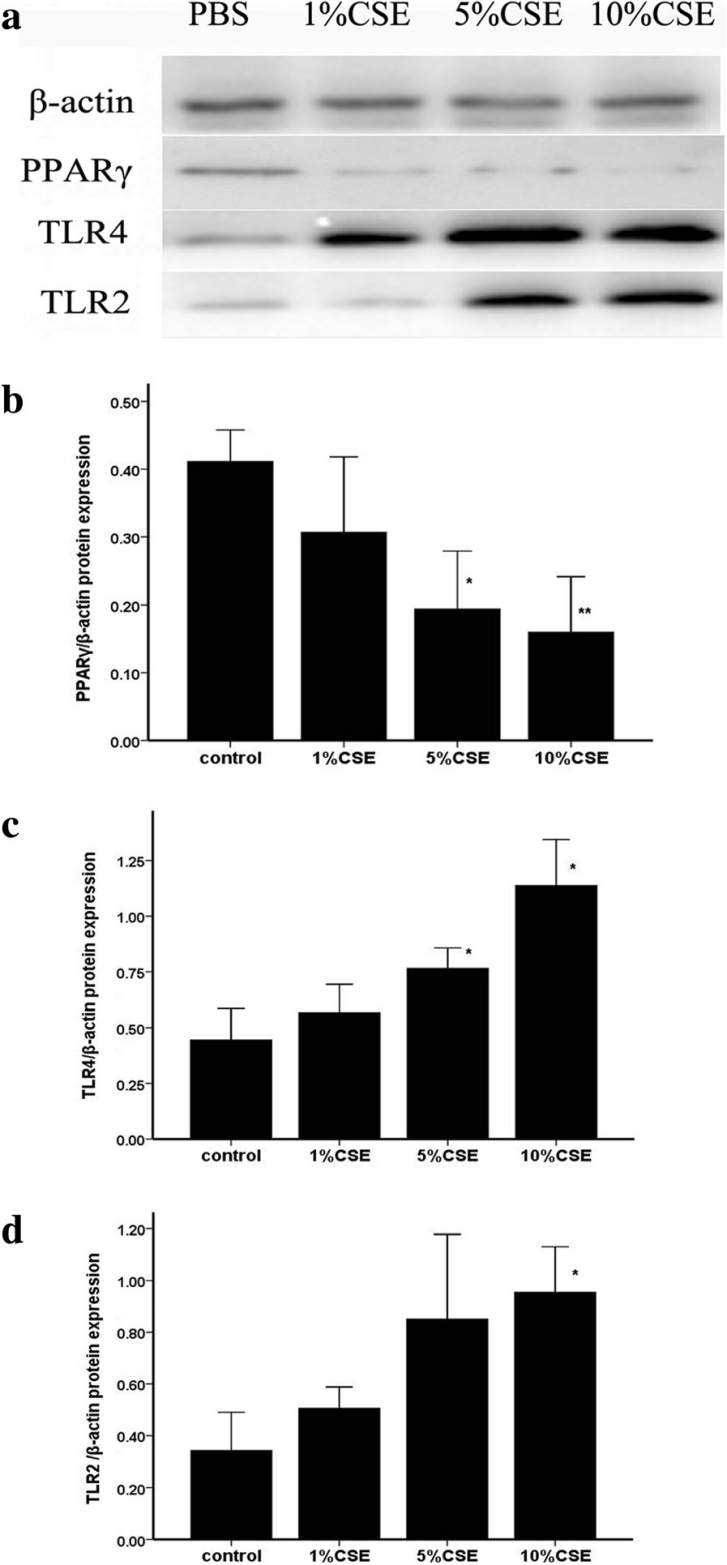
**The protein expressions of TLR2, TLR4 and PPARγ in AMs.** AMs extracted from normal rats and were treated with different concentrations of CSE for 12 hrs *in vitro*. The protein expression was determined by western blotting. **a**: Representative Western blot of TLR2, TLR4, PPARγ and actin. Image analysis of PPARγ **(b)**, TLR4 **(c)** and TLR2 **(d)** determined by densitometry. The results are expressed as the mean ± SD (n = 3). *P < 0.05 and **P < 0.01 compared with the control group.

**Figure 9:**
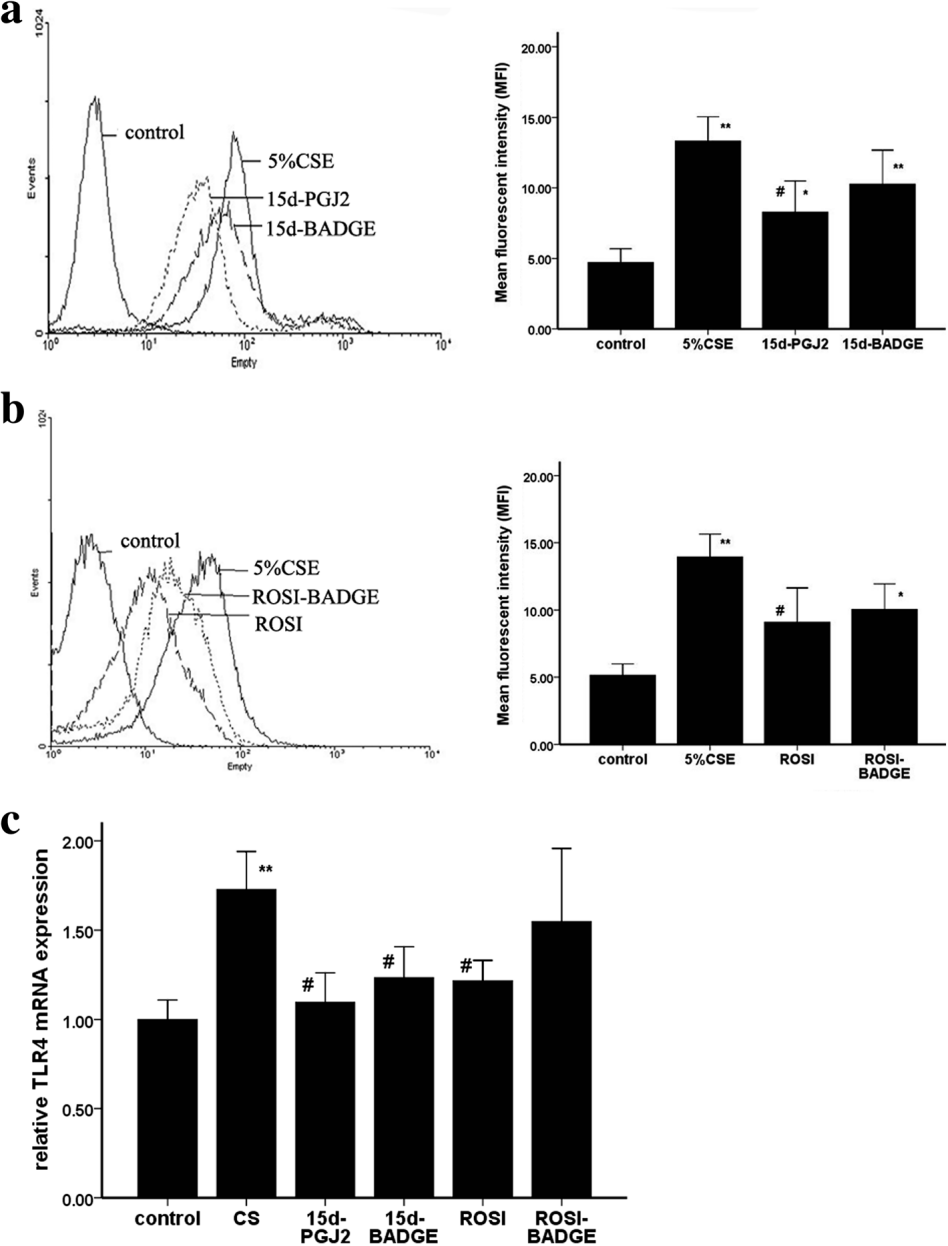
**The effect of 5% CSE on TLR4 expression *in vitro*.** The results are expressed as the mean ± SD (n = 4). **a** and **b**: representative flow cytometry histogram showing TLR4 expression on AMs treated with 5% CSE for 12 hrs. Representative flow cytometry histogram showing TLR4 expression on AMs. **c**: the expressions of mRNA of TLR4 in AMs. The mRNA was determined by real-time PCR. *P < 0.05 and **P < 0.01 compared with the CSE-exposed group.

**Figure 10:**
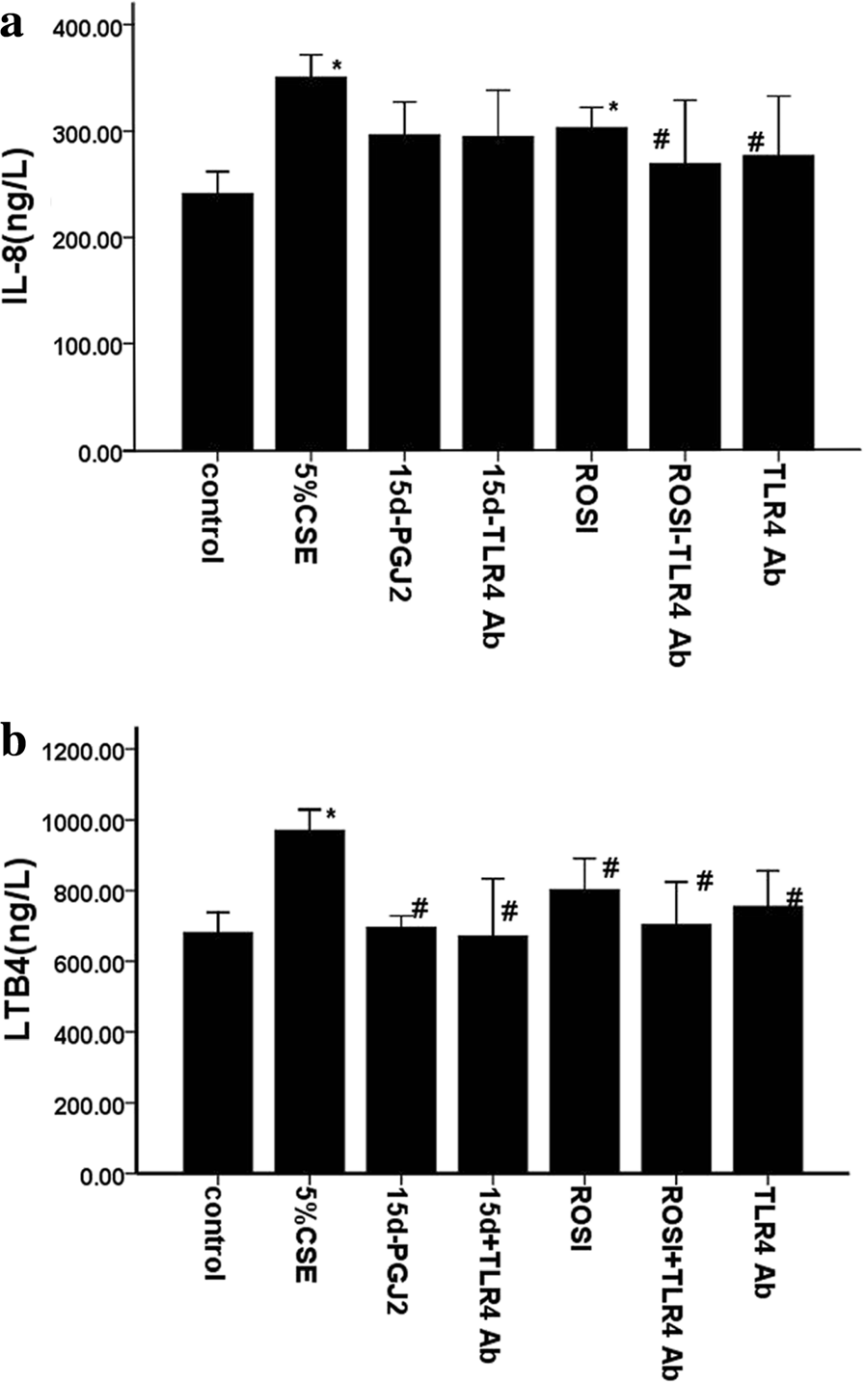
**Cell Supernatants were harvested to measure IL-8 (a)and LTB4 (b) by ELISA.** The results are expressed as the mean ± SD (n = 4). *P < 0.05 and **P < 0.01 compared with the sham group; #*P* < 0.05 and ##*P* < 0.01 compared with the 5% CSE group.

We investigated whether TLR4 were involved in CS-induced increases of inflammatory cytokines. We found that CS exposure increased TLR4 expression (Figures 6, 7, 8 and 9) as well as IL-8 and LTB4 levels *in vivo* and *in vitro*. And pretreatment with neutralizing TLR4 antibody (10 ug/ml) deduced the releases of IL-8 and LTB4 induced by 5% CSE (Figure 10). The effect of neutralizing TLR4 antibody on cytokines was similar to that of 15d-PGJ2 or ROSI. Compared to treatment with 15d-PGJ2 or ROSI alone, there was a trend toward a reduction of LTB4 (695.6 ± 52.84 vs 671.1 ± 162.22, 802.4 ± 88.76 vs 754.9 ± 101.27) and IL-8 (296.7 ± 30.38 vs 294.9 ± 43.30, 303.4 ± 38.68 vs 269.6 ± 59.10) in presence of neutralizing TLR4 antibody, but none of differences were statistical significance.

### PPARγ ligands attenuated the expression of TLR4 at message and protein levels as well as cell surface level in AMs

**Figure 11:**
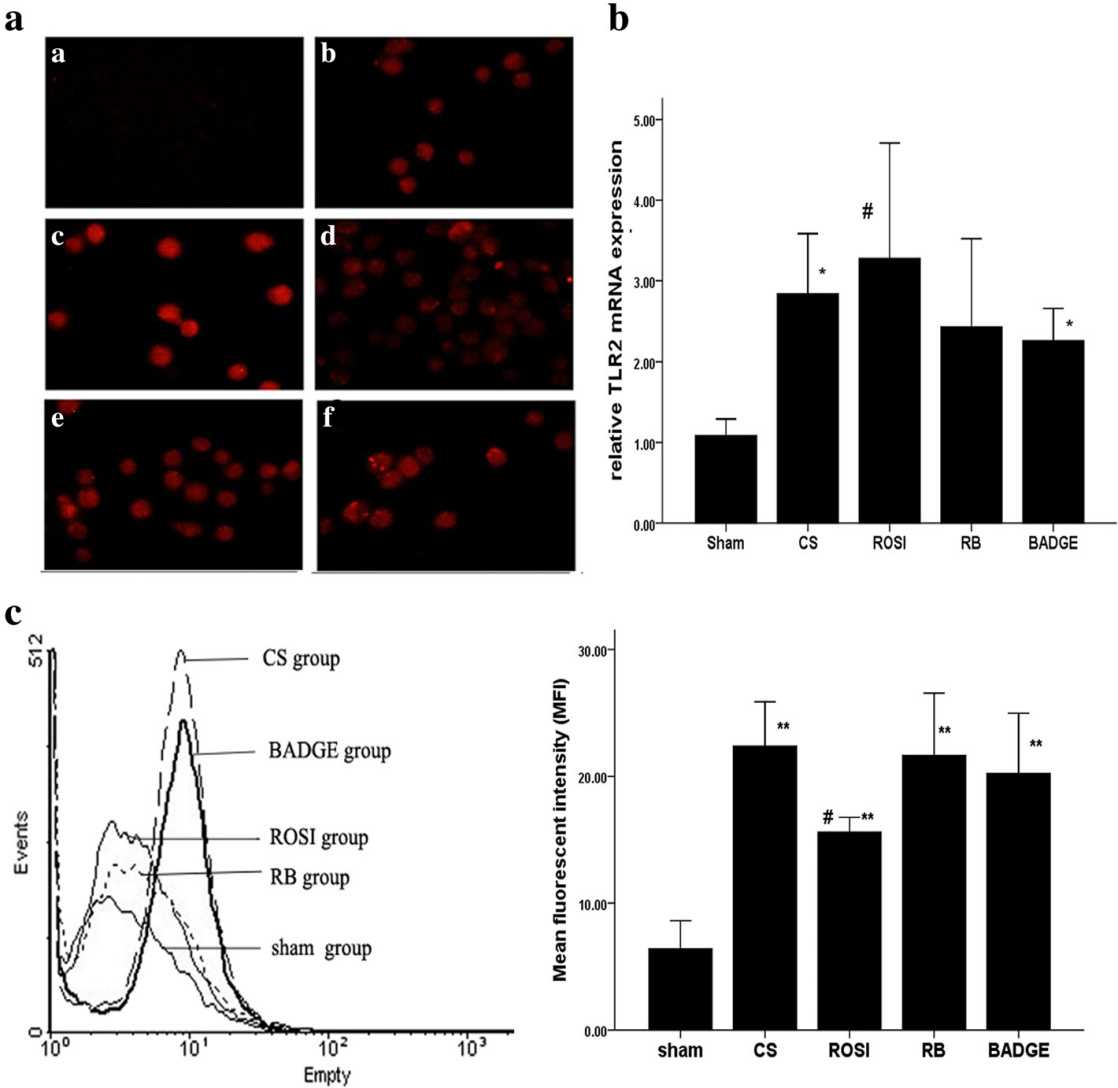
**Expression of TLR2 in AMs gained from each group. a**: Immunofluorescence of AMs from each group of rats with antibodies to TLR2 (red). Representative TLR2 expression was shown in the negative control group (a), the sham (b), CS (c), ROSI (d), RB (e) groups and BADGE (f). Shown are representative images of five rats in each group; **b**: mRNA expression of TLR2 in AMs. The data are representative of six rats in each group. **c**: representative flow cytometry histogram showing surface protein expression of TLR2 in AMs. The data are representative of six rats in each group. The results are presented as mean ± SD. *P < 0.05 and **P < 0.01 compared with the sham group; #*P* < 0.05 and ##*P* < 0.01 compared with the CS group.

**Figure 12:**
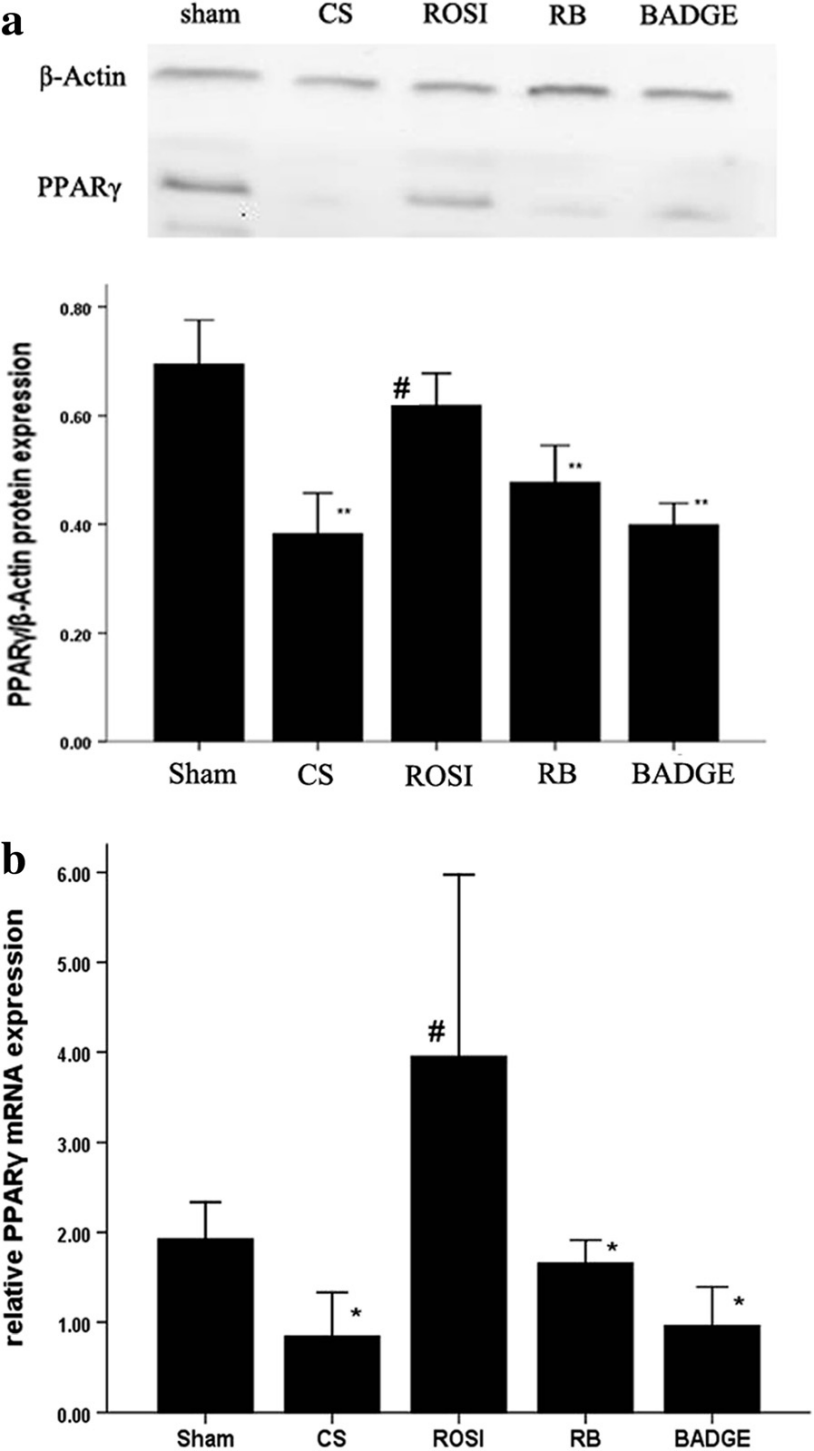
**Expression of PPARγ in AMs gained from each group. a**: protein expression of PPARγ in AMs. **b**: mRNA expression of PPARγ in AMs. The data are representative of 6 rats in each group. The results are presented as mean ± SD (n = 6). *P < 0.05 and **P < 0.01 compared with the sham group; #*P* < 0.05 and ##*P* < 0.01 compared with the CS group.

We found that CS downregulated PPARγ expression while upregulated TLR2 and TLR4 expression in AMs *in vivo*. Compared to CS exposure, treatment with ROSI increased the expressions of PPARγ, but decreased the expression of TLR2 and TLR4 *in vivo* (Figures 6, 11 and 12). Similar to the *in vivo* study, 5% and 10% CSE decreased mRNA and protein expressions of PPARγ, while increased mRNA, protein expressions and surface levels of TLR4 *in vitro* (Figures 7 and 8). Pretreatment with either ROSI or 15d-PGJ2 attenuated the mRNA and surface levels of TLR4 (but not TLR2) induced by 5% CSE (Figure 9 and Additional file 1: Figure S4), and the effects of the PPARγ agonists were blocked by treatment with BADGE *in vitro*.

**Figure 13:**
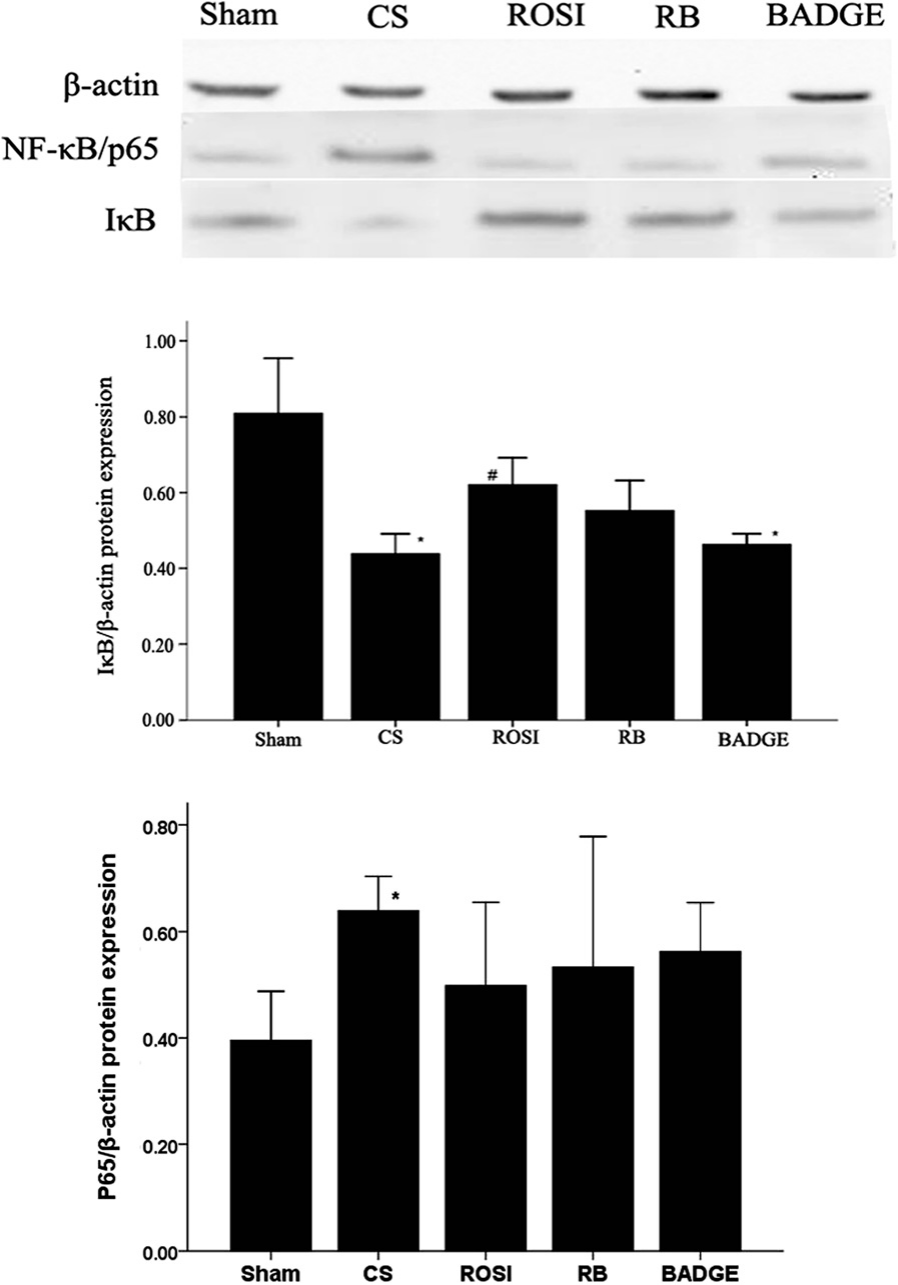
**Western blot analysis of IκBα degradation and P65 expression in AMs.** The data are representative of 6 rats in each group. The cells were harvested from each group. Four more experiments gave similar results. *P < 0.05 and **P < 0.01 compared with the sham group. #*P* < 0.05, ##*P* < 0.01, compared with the CS group.

**Figure 14:**
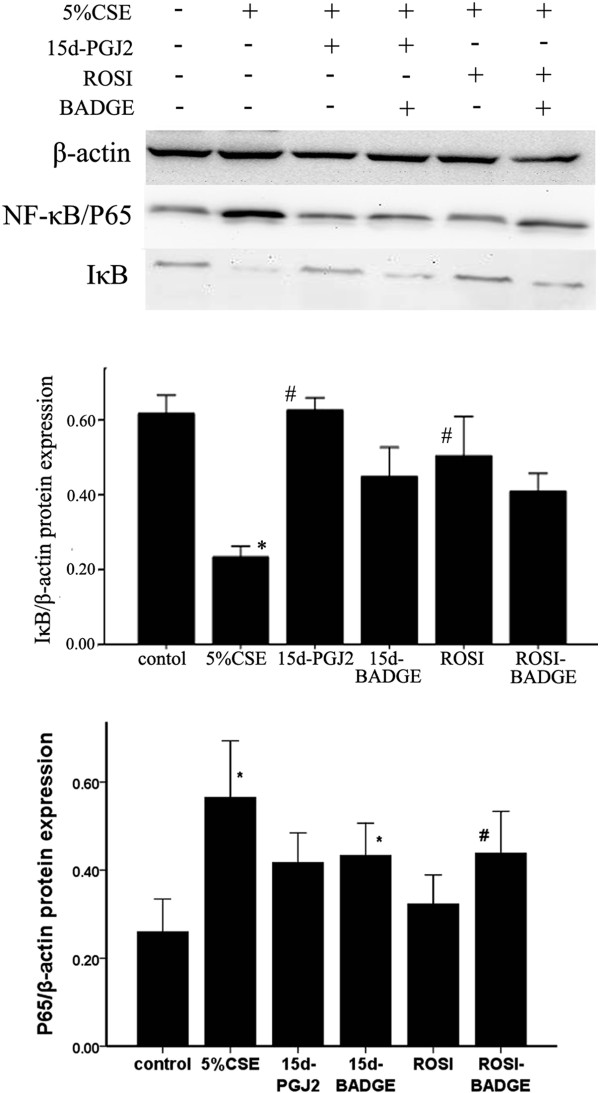
**15d-PGJ2 or ROSI stimulation delayed CSE-induced IκBα degradation *in vitro *(n = 3).** *P < 0.05 and **P < 0.01 compared with the sham group; #*P* < 0.05 and ##*P* < 0.01 compared with the CS only-exposed group.

**Figure 15:**
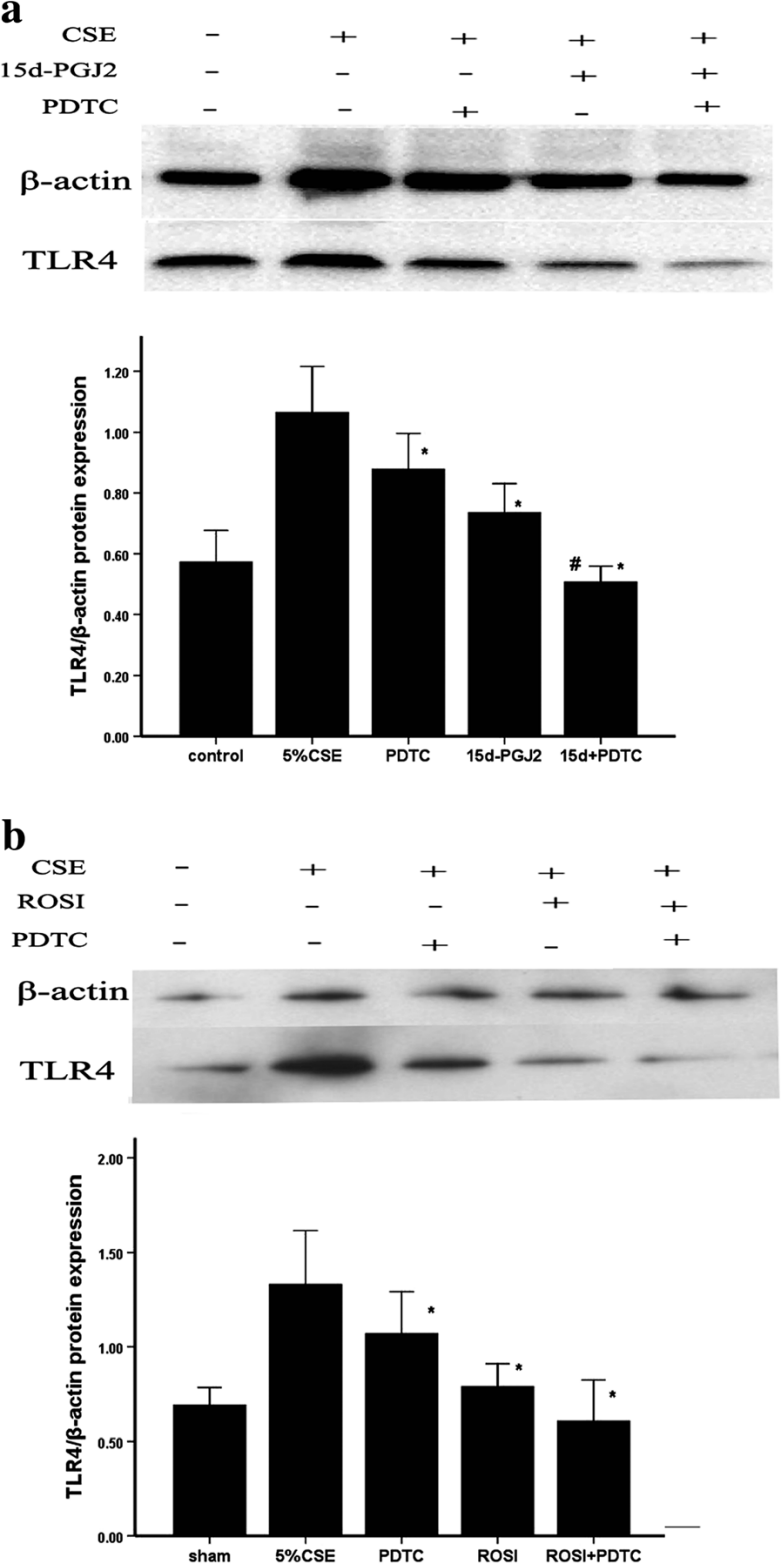
**The effect of PDTC on the PPARγ-mediated inhibition of TLR4 *in vitro *(n = 3). a** The AMs in response to 15d-PGJ2 and PDTC pretreatment interventions. **b** The AMs in response to ROSI and PDTC pretreatment interventions. *P < 0.05 and **P < 0.01 compared with the CS only-exposed group; #*P* < 0.05 and ##*P* < 0.01 compared with the PPARγ ligands (15d-PGJ2 and ROSI) group.

We next investigated whether NF-κB were involved in PPARγ mediated inhibition of TLR4. CS enhanced IκBα degradation and increased P65, TLR4 in AMs *in vivo* and *in vitro*. And pretreatment with PPARγ ligands (ROSI or 15d-PGJ2) decreased IκBα degradation, and inhibited TLR4 expressions induced by CS (Figures 13 and 14). We also found that NF-κB inhibitor PDTC reversed the increases of TLR4 induced by 5% CSE (Figure 15).

## Discussion

Cigarette smoking is a major factor influencing ongoing inflammation in the airways and lung parenchyma, with the severity of airflow limitation being correlated with the degree of pulmonary inflammation. Cigarette smoke causes airway inflammation by activating macrophages, neutrophils, and T lymphocytes. As the first line of defense against inhaled constituents, AMs are directly involved in the secretion of cytokines, including IL-8 and LTB4, and the degradation of the extracellular matrix, and can enhance emphysema [18-20]. AMs are thought to be the main orchestrators of the chronic inflammatory response and tissue destruction observed in COPD patients [21]. Similarly, our studies observed that exposure to cigarette smoke induced emphysema (data shown in the Additional file 1), while increased the total cells number counts and number of AMs in BAL fluid, decreased AMs phagocytosis and AMs viability, and increased IL-8 and LTB4 releases by AMs *in vivo* and *in vitro*. Thus, AMs were thought to be a main component of the inflammatory response to cigarette smoke.

The nuclear hormone receptor PPARγ plays an important role in a diverse range of biological processes, including the prevention of acute inflammation. Peroxisome proliferator-activated receptors (PPARs) exert anti-inflammatory effects in several cell types, such as smooth muscle cells, endothelial cells, and macrophages. Several studies have demonstrated that the *in vivo* administration of PPARγ ligands inhibited adjuvant-induced arthritis, colitis, and atherosclerosis in animal models [22-24], raising the possibility that PPARγ might be a critical component of the inflammatory process. Strong expression of PPARγ was seen in freshly isolated human AMs. It had been shown in mouse and human that PPARγ deletion from AMs was associated with resolution of inflammation and airway immunity [25], and PPAR-γ ligands upregulated phagocytosis of AMs [26,27]. The findings above suggested that it may be an protective function of PPAR-γ agonists in promoting inflammation resolution in AMs.

Some researchs showed that PPARγ expression levels was reduced in lungs of patients with moderate and severe COPD [28], in macrophages gained from BALF of COPD patients when stimulated with IFN-γ [29], and in the skeletal muscle of COPD patients [30], whereas it was increased in the lungs of rats which treated with CS + Lipopolysaccharides (LPS) and patients with mild COPD. Conversely, proportion of macrophages staining for PPAR-γ protein in tissue was similar in COPD patients [26]. In the present study, we investigated biological actions of PPAR-γ on cigarette smoke induced pulmonary inflammation in AMs. We observed that CS decreased PPARγ expression in AMs *in vivo* and *in vitro.* Here, we also found that the administration of PPARγ ligands (ROSI or 15d-PGJ2) attenuated the CS-induced inflammation in AMs *in vivo* and *in vitro*: compared to CS-treatment, the decreases in pro-inflammatory cytokines, the reductions in obvious morphological changes caused by increases in an emphysema-like phenotype and totol cell number in BAL fluid, and the increases in the phagocytosis and viability of AMs. Our findings demonstrated that PPARγ had anti-inflammatory effects on CS-induced inflammation, and it might be participated in the pathogenesis of COPD.

TLR-mediated signaling might play a crucial role in CD-induced inflammatory production [31-33]. In addition, some report suggested that TLR2 and TLR4 genes are associated with (changes in) numbers of inflammatory cells as well as with decline of lung function [34]. Our results showed that the surface protein expression of TLR4, but not of TLR2, was increased in AMs as a response to CS, accompanied with increased inflammatory cytokins secretion *in vivo* and *in vitro*, confirming the results of several reports that have demonstrated changes in the expression of TLR4 in the epithelial cells and monocytes of COPD patients [35-37]. In the present study, we used neutralizing antibody for TLR4 to investigate the role of TLR4 in CS-induced inflammation in AMs. We found that CS up-regulated both TLR4 expression and IL-8 and LTB4 releases in a dose-dependent manner. And neutralizing TLR4 antibody partially suppressed the inflammatory cytokines induced by 5% CSE. These observations indicated that TLR4-mediated inflammatory signal was implicated in the CS-related inflammatory pathogenesis.

The precise mechanism by which PPARγ exerts anti-inflammatory effects in AMs is poorly understood. We explored the PPARγ signaling pathway and searched for a relationship with the TLR4 signaling pathway *in vivo* and *in vitro*. Therefore, we further investigated the effects of two different PPARγ ligands, 15d-PGJ2 and ROSI, on the expression of TLR4 *in vitro*. We found that treatment with the PPARγ ligands reversed the CS-induced increase in TLR4 expression, confirming the results of other studies that investigated colon epithelial cells [38]. In this study, the effects of PPARγ ligands on TLR4 and cytokines secretions could be partially attenuated by treatment with PPARγ antagonist (BADGE). These data suggested that the effects of 15d-PGJ2 and ROSI on the upregulations of TLR4, IL-8 and LTB4 induced by CS were partially PPARγ-dependent. Modulation of IL-8 and LTB4 production of PPARγ ligands was also studied in the presence of TLR4 inhibitor. Our data showed that neutralizing TLR4 antibody significant inhibited the cytokin production, but could not enhance the effects of PPARγ ligands on cytokine release. We speculated the reasons of this phenomenon as followed: The finite effect of CSE on TLR4 expression might induce ceiling phenomenon. Thus the effect of PPARγ ligands on TLR4 did not stack with other TLR4 inhibitor increasing effects. The founding provided additional evidence for the role of the PPARγ-TLR4 pathway in inflammation.

Previous research has indicated that CS induces the release of pro-inflammatory cytokines in the monocyte-macrophage MonoMac6 cell line by activating NF-κB [21], and NF-κB plays a crucial role in regulating many proinflammatory mediators, including TLR4 [39]. We investigated the expression of TLR4 in the presence or absence of the NF-κB inhibitor PDTC *in vitro*. We found that PDTC could significantly reduce TLR4 protein expression induced by CS, possibly via some direct inhibitory effect of blockage of NF-kB on TLR4 expression. Thus, the inhibition of NF-κB by PDTC verified the importance of the NF-κB-TLR4 pathway in CS-induced inflammation. PPARγ have been shown to interact directly with intracellular proteins and regulate signaling pathway through modifying protein function, including the inhibition of IκBα degradation and the reduction of RelA (p65) nuclear translocation. The current *in vitro* experiment showed that both 15d-PGJ2 and ROSI treatment delayed CS-induced IκBα degradation and increased P65 expression in AMs. Based on these reports and our study, it was possible that PPARγ ligands (15d-PGJ2 and ROSI) may associate with certain signaling molecules (NF-κB) in the TLR4 signaling pathway. However, the exact mechanism need further study.

Some of the limitations of our study should be acknowledged. First, the study utilized AMs from Wistar rats. It is not known whether the same results can be observed in human cells, but these findings suggest that animal model therapeutic trials for smoke-induced lesions might better predict which drugs will be effective in treating COPD if the trials include an intervention arm that starts well into the exposure period. In addition, AMs are the only cells in the myeloid lineage that contain liver-type fatty acid binding protein (L-FABP) [40]. L-FABP is necessary for the nuclear signaling of PPARγ [41]. A previous study has shown that AMs constitutively express high levels of PPAR-γ. Therefore, we investigated the function of PPARγ in AMs. AMs are known to vary from other cells, including PMs, making it difficult to ascertain whether the protective role of PPARγ is limited to AMs or not. Second, our study emphasized the expression of only one PPAR subtype, despite the anti-inflammatory effect of the other subtypes. Third, We used rosiglitazone at the lower doses that are effective in animal models [42-44]. Although, a previous study found that only higher doses of the PPARγ ligands could affect viability and systemic side effects. Our study will further investigate the relationship between the dose of the PPARγ ligands and the side effects; Finally, inflammation is a complicated, interconnected network, and our study only investigated IL-8 and LTB4 from a vast array of other cytokines.

## Conclusions

We proved that PPARγ ligands inhibits CS-induced inflammation through a PPARγ-dependent mechanism that functions downstream of TLR4 and activates NF-κb transcriptional activity. Further investigation into the mechanisms by which PPARγ regulates AM function will improve our understanding of the role of PPARγ in chronic airway inflammation.

## Abbreviations

COPD: Chronic obstructive pulmonary disease; TLR: Toll like receptor; PPAR: Peroxisome proliferator-activated receptors; BALF: Bronchoalveolar lavage; ELISA: Enzyme-linked immunosorbent assay; LTB4: Leukotriene B4; IL-8: Interleukin-8; RT-PCR: Reverse transcription-polymerase chain reaction; CSE: Cigarette smoke extrat; AMs: Alveolar macrophage; MTT: 3-(4,5-Dimethylthiazol-2-yl)-2,5-diphenyltetrazolium bromide; NF-κB: Nuclear factor kappa B; 15d-PGJ2: 15-deoxy-Delta12,14- prostaglandin J2; BADGE: Bisphenol A diglycide ether.

## Competing interests

The authors declare that they have no competing interests.

## Authors’ contributions

YY conducted the experiments and wrote the manuscript, GH and EL contributed to the molecular biological experiments and QW and JK contributed to the design and finance of the experiments. All authors read and approved the final manuscript.

## Supplementary Material

Additional file 1: Figure S1 Prime-boost protocols for the animal experiments. Male Wistar rats were randomized into five groups of 12 animals each: sham; cigarette smoking (CS) only-exposed; rosiglitazone (ROSI)-treated; BADGE-treated; and RB-treated. The rats were sacrificed 1 week after the last smoke exposure. **Figure S2.** Photomicrographs of HE-stained lung tissue from sham (a), CS only-exposed (b), ROSI-treated (c), BADGE-treated (d), and RB-treated (e). HE staining; original magnification × 100. **Figure S3.** Photomicrographs of HE-stained lung tissue from sham (a), CS only-exposed (b), ROSI-treated (c), BADGE-treated (d), and RB-treated (e). HE staining; original magnification × 400. **Figure S4.** The effect of 5% CSE on TLR2 expression in vitro. The results are expressed as the mean ± SD (n = 4). Figure 10a and Figure 10b: representative flow cytometry histogram showing TLR2 expression on AMs treated with 5% CSE for 12 hrs. Representative flow cytometry histogram showing TLR2 expression on AMs. Figure 10c: the expressions of mRNA of TLR2 in AMs. The mRNA was determined by real-time PCR. *P < 0.05 and **P < 0.01 compared with the CSE-exposed group. **Table S1.** Morphometric results (mean linear intercept [MLI] and mean alveolar numbers [MAN]) in different groups. **Table S2.** Pulmonary function in CS group and Sham group. Click here for file
