# Supplementary material for: PPAR Gamma agonists regulate tobacco smoke-induced toll like receptor 4 expression in alveolar macrophages
Source: Respir Res. 2014 Mar 11;15(1):28. doi: 10.1186/1465-9921-15-28 (PMC4007599; doi:10.1186/1465-9921-15-28)
Supplement: Additional file 1: Figure S1 — Prime-boost protocols for the animal experiments. Male Wistar rats were randomized into five groups of 12 animals each: sham; cigarette smoking (CS) only-exposed; rosiglitazone (ROSI)-treated; BADGE-treated; and RB-treated. The rats were sacrificed 1 week after the last smoke exposure. Figure S2. Photomicrographs of HE-stained lung tissue from sham (a), CS only-exposed (b), ROSI-treated (c), BADGE-treated (d), and RB-treated (e). HE staining; original magnification × 100. Figure S3. Photomicrographs of HE-stained lung tissue from sham (a), CS only-exposed (b), ROSI-treated (c), BADGE-treated (d), and RB-treated (e). HE staining; original magnification × 400. Figure S4. The effect of 5% CSE on TLR2 expression in vitro. The results are expressed as the mean ± SD (n = 4). Figure 10a and Figure 10b: representative flow cytometry histogram showing TLR2 expression on AMs treated with 5% CSE for 12 hrs. Representative flow cytometry histogram showing TLR2 expression on AMs. Figure 10c: the expressions of mRNA of TLR2 in AMs. The mRNA was determined by real-time PCR. *P < 0.05 and **P < 0.01 compared with the CSE-exposed group. Table S1. Morphometric results (mean linear intercept [MLI] and mean alveolar numbers [MAN]) in different groups. Table S2. Pulmonary function in CS group and Sham group. [file 1465-9921-15-28-S1.docx]

**Additional files**

**Animals and experimental design**

The sham group received normal room air. Rats in the CS only-exposed group were exposed to smoke, using the homemade device (glass case, volume: 1250mm ×800mm×1200 mm, small latticed breathing hole on the side of device:1.5 cm x 1.5 cm). The rats were exposed to smoke from 16 commercial cigarettes (each cigarette contained 0.9 mg nicotine, 14 mg CO, and 12 mg tar oil; v/Vof smokescope was 8%) for 30min twice per day, 6 days per week for 12 weeks. Rats in the other three groups were exposed to CS for 12 weeks as well as pretreated with rosiglitazone (Sheng-jitang Co Ltd, Guiyang, Guizhou, China, 3mg/kg, intra-peritoneal administration), BADGE (Sigma-Aldrich Corporation, St. Louis, MO, USA, 30 mg/kg, intra-gastric administration), or both rosiglitazone and BADGE. The rats were sacrificed 1 week after the last smoke exposure.

**CS induced histological and functional changes**

When the rats exposed to cigarette smoke, their functional changes had to be appeared, as shown in their behavior and changes in pulmonary function. Smoking group rats showed that yellow rats hair, decreased activity, lack of appetite and weight gain slower. CS only-exposed group also displayed that pulmonary function in shown to decreases of FEV0.3 / FVC × 100% (data were shown in table S2 in additional files).


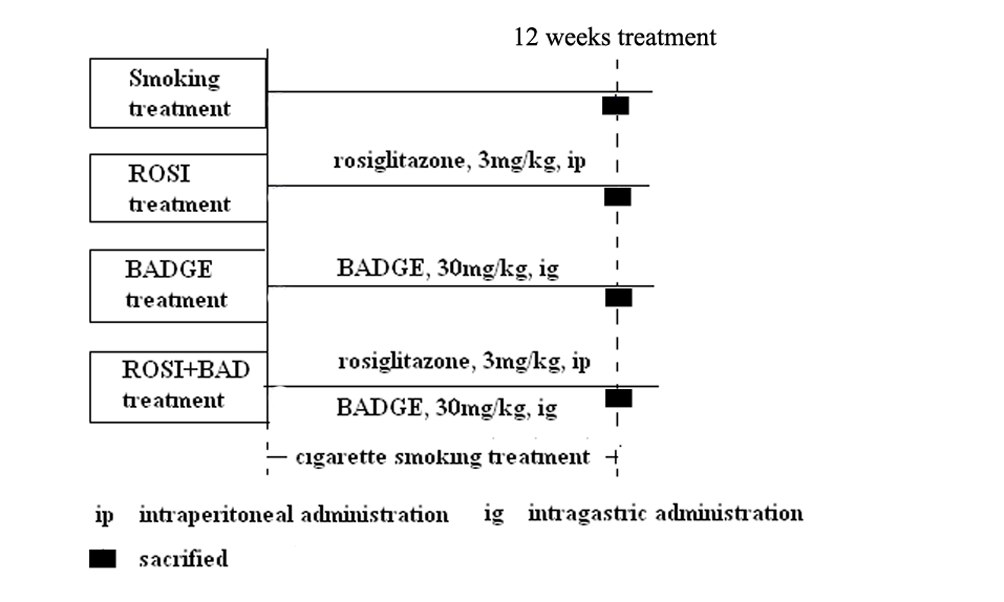


**Figure S1** Prime-boost protocols for the animal experiments. Male Wistar rats were randomized into five groups of 12 animals each: sham; cigarette smoking (CS) only-exposed; rosiglitazone (ROSI)-treated; BADGE-treated; and RB-treated. The rats were sacrificed 1 week after the last smoke exposure.


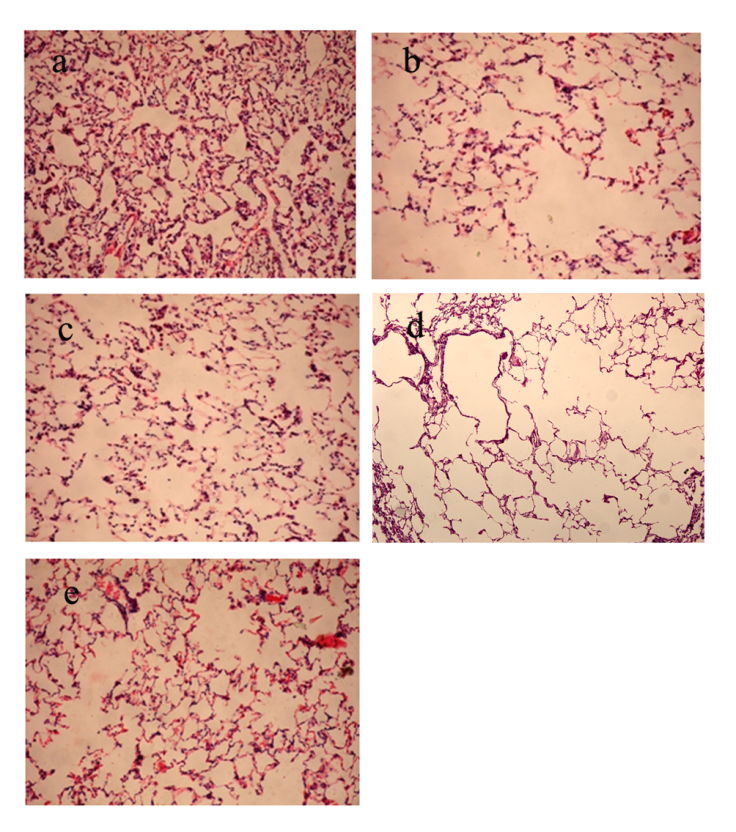


**Figure S2** Photomicrographs of HE-stained lung tissue from sham (a), CS only-exposed (b), ROSI-treated (c), BADGE-treated (d), and RB-treated (e). HE staining; original magnification × 100.

**Table S1** Morphometric results (mean linear intercept [MLI] and mean alveolar numbers [MAN]) in different groups

| Group | MLI  (um) | MAN  (units/um^2^) | |
| --- | --- | --- | --- |
| Sham | 32.2 ± 4.60 | | 16.4 ± 2.97 |
| CS only-exposed | 98.1 ± 47.94** | 4.7 ± 1.07** | |
| ROSI-treated | 49.5± 23.04* ## | 9.6 ± 2.17**## | |
| BADGE-treated | 88.1 ± 63.15** | 5.2 ± 2.00** | |
| RB-treated | 75.8 ± 42.88** | 6.2± 1.93** | |

The data represent the mean ± SD (*n* = 6). **P* < 0.05 and ***P* < 0.01 compared with sham group; #*P* < 0.05 and ##*P* < 0.01 compared with the CS only-exposed group.


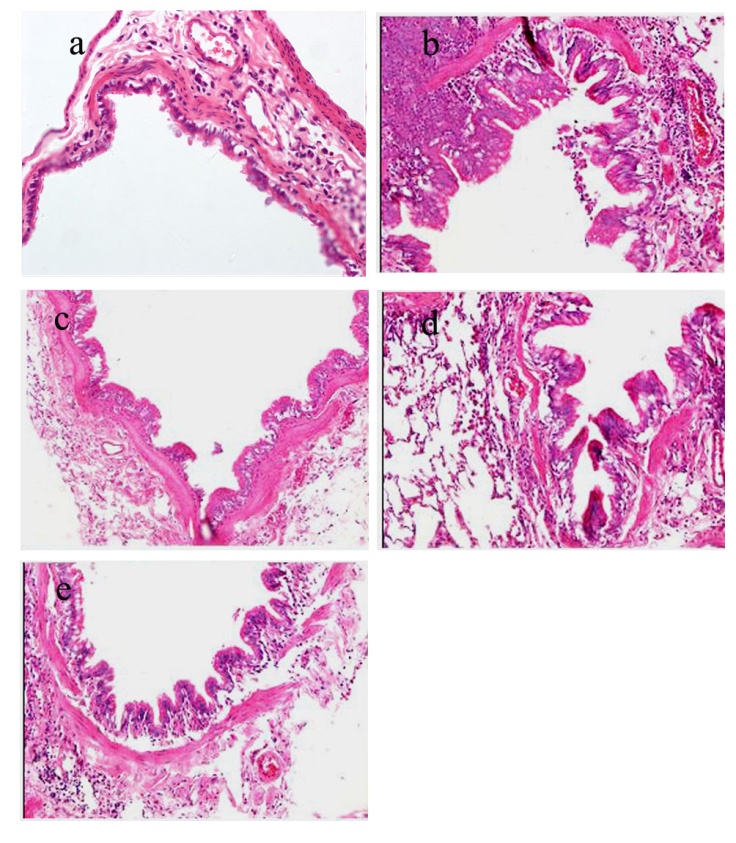


**Figure S3** Photomicrographs of HE-stained lung tissue from sham (a), CS only-exposed (b), ROSI-treated (c), BADGE-treated (d), and RB-treated (e). HE staining; original magnification × 400.

| **Table S2** Pulmonary function in CS group and Sham group | | | | | | | |
| --- | --- | --- | --- | --- | --- | --- | --- |
| **group** | **n** | **FVC**  **(ml)** | **FEV_0.3_**  **(ml)** | **FEV_0.3_/FVC**  **(%)** | **FEF_25-75_**  **(ml/s)** | **MMF**  **(ml/s)** | **PEF**  **(ml/s)** |
| **Sham** | 5 | 7.62±0.21 | 6.79±0.06 | 89.20±2.89 | 26.69±0.57 | 26.69±0.57 | 32.41±0.46 |
| **CS only-exposed** | 5 | 7.89±0.88 | 5.98±0.44 | 75.72±5.67* | 25.16±1.86 | 25.16±1.86 | 29.31±1.75* |
| The data represent the mean ± SD. **P* < 0.05 and ***P* < 0.01 compared with sham group. | | | | | | | |


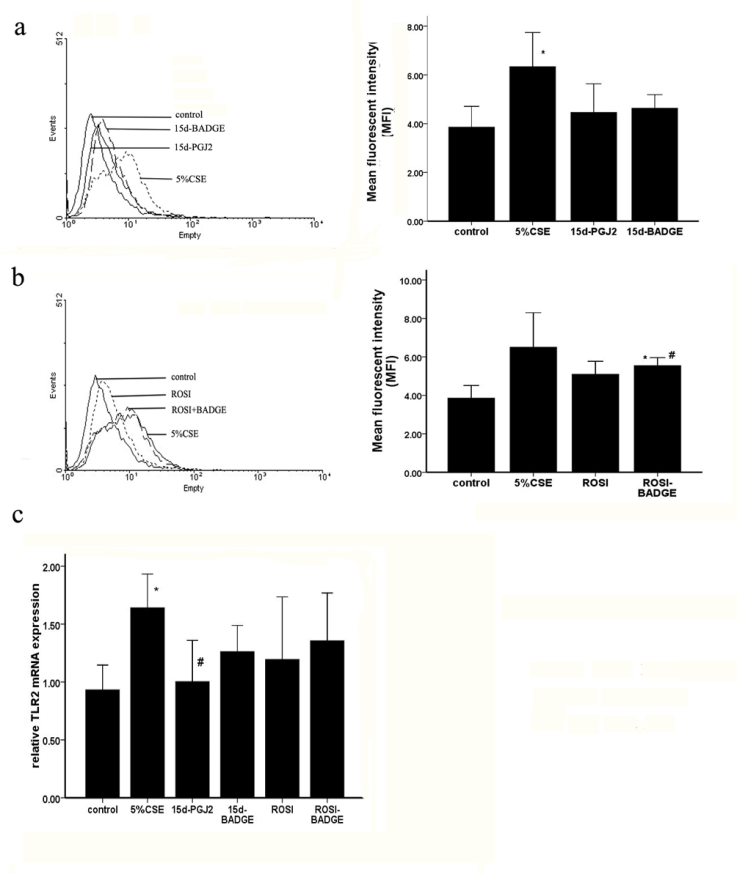


**Figure S4.** The effect of 5%CSE on TLR2 expression *in vitro*. The results are expressed as the mean ± SD (n = 4). Figure 10a and Figure 10b: representative flow cytometry histogram showing TLR2 expression on AMs treated with 5%CSE for 12

hrs. Representative flow cytometry histogram showing TLR2 expression on AMs. Figure 10c: the expressions of mRNA of TLR2 in AMs. The mRNA was determined by real-time PCR. **P* < 0.05 and ***P* < 0.01 compared with the CSE-exposed group.
